# Non-alcoholic fatty liver disease (NAFLD): a review of pathophysiology, clinical management and effects of weight loss

**DOI:** 10.1186/s12902-022-00980-1

**Published:** 2022-03-14

**Authors:** Sjaak Pouwels, Nasser Sakran, Yitka Graham, Angela Leal, Tadeja Pintar, Wah Yang, Radwan Kassir, Rishi Singhal, Kamal Mahawar, Dharmanand Ramnarain

**Affiliations:** 1 Department of Intensive Care Medicine, Elisabeth-Tweesteden Hospital, Hilvarenbeekseweg 60, P.O. Box 90151, 5000 LC Tilburg, The Netherlands; 2 Department of Surgery, Holy Family Hospital, Nazareth, Israel, and the Azrieli Faculty of Medicine, Bar-Ilan University, Safed, Israel; 3 Faculty of Health Sciences and Wellbeing, University of Sunderland, Sunderland, UK; 4 Facultad de Psycologia, Universidad Anahuac Mexico, Mexico City, Mexico; 5 Department of Bariatric Surgery, Christus Muguerza Conchita Hospital, Monterrey, Mexico; 6 Department of Abdominal Surgery, University Medical Center Ljubljana, Zaloška cesta, Ljubljana, Slovenia; 7 Department of Metabolic and Bariatric Surgery, The First Affiliated Hospital of Jinan University, Guangzhou, China; 8 CHU Félix Guyon, Allée des Topazes, Saint-Denis, France; 9 Bariatric and Upper GI Unit, Birmingham Heartlands Hospital, University Hospital Birmingham NHS Foundation Trust, Birmingham, UK; 10 Bariatric Unit, South Tyneside and Sunderland NHS Foundation Trust, Sunderland, UK; 11 Department of Intensive Care Medicine, Saxenburg Medical Centre, Hardenberg, The Netherlands

**Keywords:** NAFLD, Non-alcoholic fatty liver disease, Weight management, Bariatric surgery, Metabolic surgery, Conservative therapy

## Abstract

Given the increasing prevalence of diabetes and obesity worldwide, the deleterious effects of non-alcoholic fatty liver disease (NAFLD) are becoming a growing challenge for public health. NAFLD is the most common chronic liver disease in the Western world. NAFLD is closely associated with metabolic disorders, including central obesity, dyslipidaemia, hypertension, hyperglycaemia and persistent abnormalities of liver function tests.

In general NAFLD is a common denominer for a broad spectrum of damage to the liver, which can be due to hepatocyte injury, inflammatory processes and fibrosis. This is normally seen on liver biopsy and can range from milder forms (steatosis) to the more severe forms (non-alcoholic steatohepatitis (NASH), advanced fibrosis, cirrhosis and liver failure). In these patients, advanced fibrosis is the major predictor of morbidity and liver-related mortality, and an accurate diagnosis of NASH and NAFLD is mandatory. Histologic evaluation with liver biopsy remains the gold standard to diagnose NAFLD. Diagnosis of NAFLD is defined as presence of hepatic steatosis, ballooning and lobular inflammation with or without fibrosis. Weight loss, dietary modification, and the treatment of underlying metabolic syndrome remain the mainstays of therapy once the diagnosis is established. Dietary recommendations and lifestyle interventions, weight loss, and the treatment of underlying metabolic syndrome remain the mainstays of therapy once the diagnosis is established with promising results but are difficult to maintain. Pioglitazone and vitamin E are recommended by guidelines in selected patients. This review gives an overview of NAFLD and its treatment options.

## Background

Non-alcoholic fatty liver disease (NAFLD) is a common cause of chronic liver disease worldwide. NAFLD is a spectrum of the disease characterized by hepatic steatosis when no other causes for secondary hepatic fat accumulation (e.g., excessive alcohol consumption) can be identified. NAFLD ranges from the more benign condition of non-alcoholic fatty liver (NAFL) to non-alcoholic steatohepatitis (NASH), which is at the more severe end of the spectrum. NAFLD may progress to fibrosis and cirrhosis [1, 2]. In NAFLD, hepatic steatosis is present without evidence of inflammation, whereas in NASH, hepatic steatosis is associated with lobular inflammation and apoptosis that can lead to fibrosis and cirrhosis [1–4].

Before the middle of the last decade, NASH was widely considered a serious condition, occurring almost exclusively in females with obesity, often associated with Type 2 Diabetes Mellitus (T2DM), and relatively benign prognosis, that are predictive risk factors of cardiovascular disease, stroke, and diabetes [1–4]. The prevalence of liver disease (NAFLD) has risen rapidly in Western countries, with a worldwide prevalence of 25%. NAFLD is becoming more common chronic liver disease in Western industrialized countries, particularly in patients with central obesity, T2DM, dyslipidaemia, and metabolic syndrome [5].

In terms of diagnostic tests, the gold standard to investigate any form of liver inflammation e.g. damage is a liver biopsy. In the diagnosis of NAFLD and related disorders, liver biopsies can be extremely helpful and its findings can range from triglyceride deposition as droplets in the hepatocyte to more extensive forms of non-alcoholic steatohepatitis (NASH). NASH is normally characterised by the earlier mentioned lipid droplets in hepatocytes, with concomitant inflammation and a variable degree of hepatic fibrosis. In the majority of the liver steatosis patients, the disease is ‘non-progressive’, however a small portion of these patients develop the earlier mentioned NASH, which can lead to liver failure and even hepatocellular carcinoma [5, 6].

NAFLD management’s US guidelines define NAFLD as steatosis with ≥5% fat infiltration in imaging or histology and b) no alcohol, drug, or viral-induced steatosis. NAFLD patients may present with elevated liver enzymes [6].

Patients with NAFLD often have one or more components of the metabolic syndrome (MS) like systemic hypertension, dyslipidaemia, Insulin resistance, or overt diabetes [7]. There is increasing evidence that visceral obesity is a risk factor for NAFLD and it also has to be taken into account that MS is a known risk factor in cardiovascular disease development [5, 7]. Based on current literature cardiac and vascular diseases seem to be the most important cause of death in these patients, however the pathophysiological mechanisms connecting cardiovascular disease and NAFLD are not fully understood [5, 7]. It is thought that insulin resistance is a common factor in the pathogenesis linking both entities [5, 7].

Evaluation of abnormal liver enzyme levels in an otherwise healthy patient can pose a challenge to even an experienced clinician. NAFLD is a common explanation for abnormal liver test results in blood donors. It determines asymptomatic elevation of alanine aminotransferase (ALT) and asparate aminotransverase (AST) levels in up to 90% of cases, once other liver disease causes are excluded [8, 9]. The World Health Organization Global Health Observatory data in 2014 indicates that globally obesity occurs in 15% of women and 11% of men aged 18 and over [8, 9]. A study evaluating the prevalence of NASH estimated that 5.7–17% of the US population is affected [8, 9].

The spectrum of NAFLD and related disease, including its complex relationship with MS poses challenges for clinicians. This review aims to summarise the pathophysiology of NAFLD, risk factors, diagnostic measures and conservative and surgical treatment options.

### Pathophysiology of NAFLD/NASH

The development of NASH is a complex process and is not completely understood. In the recent years a lot of animal research has been conducted investigating the pathophysiology of NAFLD and NASH, mainly difference in dietary models (high fructose, high fat or methionine/choline deficient diet (MCD) [10, 11]. Based on this body of evidence, it has been suggested that the development of NASH is a two-step process. The first step of this process is fat deposition in the liver that will increase insulin resistance. The second part of this process is cellular and molecular changes involving oxidative stress, and oxidation of fatty acids in the liver due to a variety of factors (cytokine injury, hyperinsulinemia, hepatic iron and/or lipid peroxidation, variation of the extracelular matrix, energy homeostasis, and change in the immune system function) [9, 12]. The development of insulin resistance is an intricate process. In the setting of the MS, as is the case for many patients with NASH, the increase in fat mass and adipocyte differentiation plays a key role in developing insulin resistance.

NAFLD can be divided into two distinct types. The first type of NAFLD has a narrow relationship with metabolic syndrome and the current beliefs are that insulin resistance is the primary pathophysiological mechanism. The second type of NAFLD has a relationship with infectious pathologies that can lead to the occurrence of liver steatosis. In this case infections like hepatitis C and HIV can be a cause, but it is also associated with medication (total parenteral nutrition, glucocorticoids, tamoxifen, tetracycline, amiodaron, methotrexate, valproic acid, vinyl chloride) and specific toxins or inherited/acquired metabolic diseases (e.g. lipodystrophy or cachexia or intestinal bypass surgery) [13, 14].

### Risk factors

People with NAFLD generally have characteristics of MS, with the associated cardiovascular disease risk factors [15, 16]. As stated earlier NAFLD is closely related to metabolic syndrome and obesity, type 2 diabetes mellitus (T2DM), and dyslipidaemia are considered to be important risk factors for NAFLD [17]. Studies have shown the increased prevalence of cardiovascular disease (CVD) in patients with NAFLD, without and without diabetes [18, 19]. Thus, NAFLD is generally associated with an unhealthy lifestyle, with evidence, to suggest that changes in unhealthy lifestyles can reduce the transaminase levels and improve NAFLD [20]. A study of patients with T2DM found a higher prevalence of peripheral vascular, coronary and cerebrovascular diseases in subjects with NAFLD than without, with coronary, cerebrovascular and peripheral vascular disease was greater among those with NAFLD than among those without this disease, aside from normal CVD risk factors, medication use and diabetes-related variables [21]. Byrne et al. stated there are over 20 published studies, both prospective and retrospective, that have studied the relationship between NAFLD and cardiovascular disease and concluded that CVD is a clear and present threat [15], which continues to be confirmed in on-going studies [22].

The relationship between NAFLD and smoking is controversial [23, 24]. Overall, smoking is a major risk factor for the development of chronic, non-communicable diseases (NCD) such as cancer, T2DM, respiratory and cardiovascular conditions globally [25]. A study of obese rats found that cigarette smoke increased the histological severity of NAFLD [26]. A cross-sectional study of NAFLD patients (smokers and non-smokers) found the proportions of patients with liver significant fibrosis and advanced liver fibrosis among the smokers were significantly higher than those among the non-smokers [20]. A systematic review and meta-analysis of 20 published studies found smoking is greatly associated with NAFLD, and recommended further studies to understand the underlying mechanisms of the association [27]. Smoking was considered an independent risk factor for the development of NAFLD [23], however, a cross-sectional study of 933 patients (368 smokers and 565 non-smokers as controls) found no difference in the prevalence of NAFLD in the two groups (22.2% versus 29%), nor with heavy smokers (> 20 packs of cigarettes per year) [28].

### Symptoms and signs

The majority of the patients with NAFLD do not experience any symptoms, however some of them may complain of fatigue, right upper quadrant discomfort, hepatomegaly, acanthosis nigricans, and lipomatosis [29]. A significant amount of patients with cirrhosis can be present themselves with end-stage liver disease. In approximately 48-100% NASH can be asymptomatic and very often it is discovered during medical evaluations for other reasons. Although clinical stigmata of chronic liver failure are rarely seen in this population, one study showed that at the time of diagnosis splenomegaly was present in 25% of the patients [29].

Very often a diagnosis like NASH or NAFLD is discovered due to abnormal liver function tests such as aminotransferases (ALT and AST) or incidental finding of hepatic steatosis on radiologic abdominal finding. Hepatomegaly can present during physical examination and this is caused by the liver’s fatty infiltration [29].

### Laboratory findings

When performing laboratory tests, serum markers like aminotransferases (AST, ALT), are mild to moderately elevated [30]. However, the AST and ALT levels can be aspecific in patients with NAFLD or related conditions. In other words, AST and ALT levels can either be elevated or normal, but both do exclude the presence of NAFLD [30–32]. In patients with NAFLD, ALT elevations are more common than elevations of AST. The ALT levels tend to be higher in NASH than in simple steatosis. Elevated serum ferritin levels are commonly elevated in patients with NAFLD, and increased transferrin saturation is found in 6–11% of patients [30–32].

Other markers of interest are alkaline phosphatase (ALP) and clotting factors. In patients with NAFLD ALP can be abnormal and even be elevated 2-3 times the upper limit of its normal value.

In addition, other laboratory values can be helpful in diagnosing NAFLD. Both albumin and bilirubin levels may be high in patients who have developed chronic progressive disease. In cirrhotic patients laboratory measurements of clotting times can be abnormal. Most of the time patients who have developed cirrhosis have a prolonged prothrombin time, thrombocytopenia, and a concomitant neutropenia [30–32].

### Imaging in NAFLD

In liver diseases such as NAFLD and NASH, various imaging modalities can be used to substantiate the diagnosis, however none of them are routinely used for differentiating between (histological) subtypes of NAFLD or NASH [33]. Computed tomography (CT) scans, abdominal ultrasound (US), or Magnetic Resonance Imaging (MRI) can detect these liver diseases. Imaging findings in patients with NAFLD include increased echogenicity on ultrasound, decreased hepatic attenuation on CT, and an increased fat signal on MRI [33].

#### Ultrasound

US often reveals a hyperechoic texture or a bright liver because of diffuse fatty infiltration [34]. The sensitivity and the specificity of US are respectively 89 and 93% in detecting increased fibrosis and steatosis [35]. However, the US is the cheapest method and has been the most common modality used in clinical practice. The sensitivity of US is decreased in patients with obesity [36, 37]. The US showing hyperechogenic liver tissue in contrast to the spleen or kidney echogenicity is suggestive of steatosis. However, the sensitivity of the US is only 60–94% in these instances [38].

#### Vibration-controlled transient Elastography (VCTE)

VCTE is a non-invasive method for excluding advanced fibrosis in measuring liver stiffness with VCTE [39–41]. A meta-analysis of 19 biopsy-controlled studies including over 2700 patients, the optimal cut-off value for steatosis grade > S0 was 248 dB/m (95% Confidence Interval (CI) 237-261) and for steatosis grade > S1 was 268 dB/m (95% CI 257-284) [40].

#### CT, MRI, and magnetic resonance spectroscopy (MRS)

Both imaging modalities are able to detect steatosis, but lack sensitivity to detect inflammatory or fibrotic process of the liver [42].

Unfortunately MRS has a higher sensitivity to detect the earlier mentioned pathological processes it is (not yet) widely available [43]. In general the sensitivity of CT, MRI and MRS to detect steatosis of the liver was 33, 50, and 88%, respectively. Specificity of all three for detection of hepatic steatosis was 100, 83, and 63%, respectively [44].

### Histological findings in NAFLD

The liver biopsy is the Gold standard for diagnosis of NASH or NAFLD. Performing a liver biopsy on every patient with suspected NAFLD remains controversial. The general indications for performing a liver biopsy in patients with NAFLD confirm or exclude the diagnosis. To many, the liver biopsy is considered to be the golden standard in diagnosing NAFLD, but a pre-emptive diagnosis is mostly made up using the medical history, laboratory work and imaging of the patient. A liver biopsy can be very helpful in assessing the amount of hepatic damage in general, but also in patients that remain to have an unclear diagnosis after non-invasive assessments [45–47].

Limitations of liver biopsy are sampling error variability, inter and intra-observer variability, and risk and complications—problems with a pathological diagnosis that may lead to misdiagnosis and staging inaccuracies. Many studies have shown sampling variability and uneven distribution of NASH histologic lesions during the evaluation of paired biopsies [45–47].

The NAFLD Activity Score (NAS) is a validated score that is used to grade disease activity in patients with NAFLD [48]. The NAS has several components and each of them has a minimum and maximum score; steatosis (0 to 3), lobular inflammation (0 to 3), hepatocellular ballooning (0 to 2) [48]. Fibrosis is not included in the NAS. In the original study that derived the NAS, scores of 0 to 2 occurred in cases mainly considered not diagnostic of NASH; scores of 3 to 4 were evenly divided among those considered not diagnostic, borderline, or positive for NASH; and scores of 5 to 8 occurred in cases that were considered mainly diagnostic of NASH [48].

### Treatment

The treatment of NAFLD and related diseases (including but not limited to components of MS) consists of several tiers of which conservative and surgical therapies are known treatments. Very often the treatment of patients with NAFLD consists of a multimodal intervention targeting multiple aspects like weight loss, lifestyle modifications and possible medication optimisation.

### Conservative treatment - lifestyle modifications and weight loss

To date, there is no specific drug treatment for NAFLD, however it is believed that a combination of treatment goals (lifestyle adjustments, increasing physical activity and smoking/ alcohol cessation) can be beneficial [49, 50].

NAFLD patients, whether living with obesity are not, should be encouraged and educated to partake in a healthy lifestyle approach, which exists irrespective of weight-loss [49]. A healthy diet i.e. reduction of caloric intake and high-glycaemic index (GI) foods, increased consumption of monounsaturated fatty acids, omega-3 fatty acids, fibers, and specific protein sources such as fish and poultry are suggested to have beneficial effects [51]. Studies suggest that a Mediterranean diet, defined as reduced carbohydrate intake (especially sugars and refined carbohydrates) and increased monosaturated and omega-3 fatty acid intake, can reduce liver fat and thus positively contribute to the management of NAFLD [50, 52]. Research into the effect of diet on risk and management of NAFLD found that prolonged consumption of sugary drinks had a positive correlation to NAFLD [53] and many people with NAFLD tended to consume high levels of sugary drinks and red meat compared to others [54]. Whilst the reduction of and type of carbohydrates on prevention and management of NAFLD has been widely studied, the effects of protein consumption are not as widely known [55, 56]. A high protein diet is suggested to be beneficial for NAFLD management, but the source of protein needs to be considered given the evidence on consumption of red meat on cardiovascular disease, and consideration of proteins in a vegetarian diet [57].

Other dietary components can also be beneficial in the treatment of NAFLD, like changes in Vitamin E, caffeine and polyphenol intake. Vitamin E is a fat-soluble vitamin that works as an antioxidant [58]. Current data support the use of Vitamin E in non-diabetic patients with nonalcoholic fatty liver disease. However, it should not be considered as the first option for treatment [58]. Vitamin E therapy should be considered as treatment if lifestyle modifications do not produce the expected results due to non-compliance or ineffectiveness [59].

The PIVENS study showed that a 2-year period of treatment with Vitamin E at 800 IU / day in adult patients significantly reverses steatohepatitis and significantly reduced hepatic steatosis and alanine aminotransferase (ALT), but did not had significant changes in fibrosis compared to placebo [58, 60]. Long-term safety is of concern, several meta-analyses suggest increased mortality in patients taking Vitamin E supplements. Some of them showed a 20% increased risk of haemorrhagic stroke; and another test suggested an increased risk of prostate cancer in men over the age of 50 [60].

Caffeine is a strong antioxidant that could help reduce the burden of oxidative stress and inflammation in the liver and may provide hepatoprotective effect [61]. Many studies have linked coffee consumption to improvement in liver enzymes in a dose-dependent manner in individuals who are at risk of liver diseases [61]. There is an evident protective role of more than 3 cups of coffee consumption per day but not less than 2 cups according a meta-analysis study [62].

Finally, polyphenols are a heterogeneous class of plant-derived compounds that include several hydro soluble antioxidants reported as health promoting agents and proposed in the treatment of different metabolic disorders [63]. Natural polyphenols are present in nature and particularly have been found in high quantities in many foods and plants, such as vegetables, fruits, cereals, spices, mushrooms, tea, microalgae, medical plants, wild fruits, and flowers [64].

There exists a considerable amount of evidence indicating the hepatoprotective effects of these biomolecules, unless they are in cultured cells and animal models. Their optimal dose and the concomitant length of the treatment period are not known [60].

### Weight loss – conservative and surgical

Weight loss is the primary therapy for most patients with NAFLD. Weight loss can improve liver biochemical tests, liver histology, serum insulin levels, and quality of life in patients with NAFLD [65–69].

A significant body of literature has shown that weight loss induces a clinical improvement in patients with NAFLD or NASH. Several studies showed improvement of liver biochemistry after significant weight loss [65–70]. In a study of 25 Japanese individuals aminotransferases, cholesterol and fasting glucose improved significantly after a 3-months of combined exercise and dieting [69]. In a study done by Knobler et al. in 48 patients these results were substantiated by the fact that the majority of the patients had improved liver biochemistry and half of them had a complete normalisation of their transaminase profiles [68].

In the last few years there have been promising outcomes using pioglitazone (and related medication) [71, 72], but also the use of Glucagon-like peptide-1 (GLP-1) receptor agonists has shown significant improvement in hepatic outcomes in patients with NAFLD [73, 74].

In the last years, bariatric and metabolic surgery has made enormous developments. Its benefits regarding weight loss and improvement of several metabolic diseases, like T2DM has been well established [75, 76]. It even has lead to a significantly better long-term overall survival compared to patients treated conservatively [75–77].

Specifically looking at NAFLD related outcomes, bariatric and metabolic surgery has not been taken into account as treatment option in many meta-analyses to date, despite the increasing body of evidence and properly designed studies on this subject. Lassailly et al. [78] found that bariatric and metabolic surgery results in resolution of NAFLD/NASH in the majority of the patients (64.2% in patients that underwent Roux-Y Gastric Bypass (RYGB) and 5.5% in Sleeve Gastrectomy (SG)). Secondly they found regression of the present liver fibrosis [78].

A meta-analysis on 48 studies showed that the combination of pioglitazone and Roux-en Y Gastric Bypass surgery had the best effects on the NAFLD Activity Score. This suggests a possible causal connection between glucose metabolism and NAFLD development [79]. It needs to be said that bariatric and metabolic surgery solely can impact the amelioration of NAFLD and related disorders, but also its impact on postoperative outcomes after surgery needs to be taken into account [80].

**Fig. 1:**
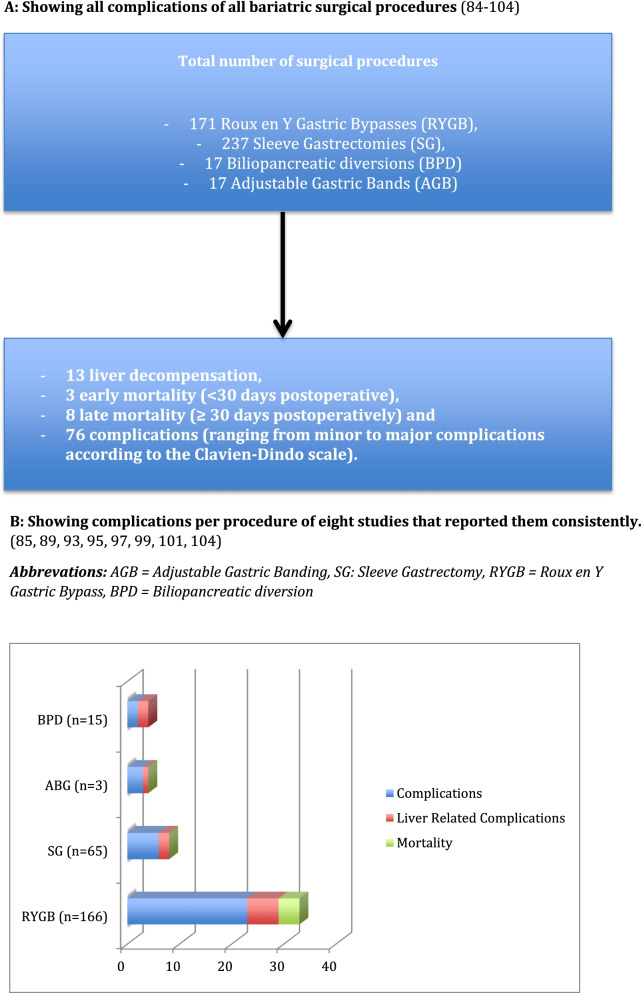
Complications in patients with liver cirrhosis undergoing bariatric and metabolic surgery. * Studies used in both the reviews by Jan et al. [82] en Ahmed et al. [83] (References [84–104]). **A** Showing all complications of all bariatric surgical procedures [84–104]. **B** Showing complications per procedure of eight studies that reported them consistently [85, 89, 93, 95, 97, 99, 101, 104]. .Abbrevations: AGB = Adjustable Gastric Banding, SG: Sleeve Gastrectomy, RYGB = Roux en Y Gastric Bypass, BPD = Biliopancreatic diversion

It has to be said that a small proportion of the patients undergoing bariatric and metabolic surgery develop NASH or suffer from aggravation of the disease (NAFLD/NASH/ live fibrosis) after bariatric surgery [81]. Also the patients on the ‘end of the spectrum’, with liver cirrhosis are in need of special preparation before and after bariatric and metabolic surgery, as highlighted in the systematic reviews done by Jan et al. [82] and Ahmed et al. [83] We need to consider that different surgical procedures have different effects on postoperative physiological remodelling and also can lead to (liver-related) complications. (See Fig. 1).

## Conclusions

With the growing obesity pandemic and the rising prevalence of comorbid conditions like T2DM and NAFLD, the management of these patients has become even more complex. There are some treatment methods, however there is lack of high-quality studies that compared different treatment methods with each other. Considering that bariatric surgery is increasingly utilized, prospective studies answering the remaining questions on the connection of insulin resistance, fatty liver, and fibrosis progression should become available in the near future.

## Data Availability

Not applicable.
